# CD34+ cells cultured in stem cell factor and interleukin-2 generate CD56+ cells with antiproliferative effects on tumor cell lines

**DOI:** 10.1186/1479-5876-3-15

**Published:** 2005-04-14

**Authors:** Giuseppe Sconocchia, Maurizio Provenzano, Katayoun Rezvani, Jongming Li, Jos Melenhorst, Nancy Hensel, A John Barrett

**Affiliations:** 1 Hematology Branch, Stem Cell Allotransplantation Section, National Hearth Lung and Blood Institute, Bethesda, MD, USA; 2 Department of Transfusion Medicine, Clinical Center, National Institutes of Health, Bethesda, MD, USA

**Keywords:** NK cells, cellular proliferation, cellular differentiation

## Abstract

In vitro stimulation of CD34+ cells with IL-2 induces NK cell differentiation. In order to define the stages of NK cell development, which influence their generation from CD34 cells, we cultured G-CSF mobilized peripheral blood CD34+ cells in the presence of stem cell factor and IL-2. After three weeks culture we found a diversity of CD56+ subsets which possessed granzyme A, but lacked the cytotoxic apparatus required for classical NK-like cytotoxicity. However, these CD56+ cells had the unusual property of inhibiting proliferation of K562 and P815 cell lines in a cell-contact dependent fashion.

## Introduction

NK cells are key mediators of innate immunity contributing to immunosurveillance by recognizing and killing tumor and virus-infected cells. They are cytolytic and produce inflammatory cytokines [1,2]. Mature NK cells are CD3-CD56+ and variably CD16+. The molecule CD56 is a 120–180 KD N-linked glycosylated isoform of the neural cell adhesion molecule (NCAM) [3]. It is expressed on NK cells, NK-T cells, and a subset of dendritic cells. NK cells originate in the bone marrow from a CD34+Lin- common lymphoid progenitor cells [4]. In the absence of bone marrow stroma, NK cell generation requires a combination of IL-2 or IL-15 [5,6] and stem cell factor (SCF) [7]. However, the early stages of CD56+ cell generation and the origin of diversification into mature CD56+ cell types are not well characterized.

We previously found that in culture with IL-2 and SCF CD34+ cells differentiate into several CD56+ subpopulations – a minor myeloid subset consisting of large CD56^dim ^CD33+ macrophage-like cells and a major lymphoid subset of CD56^bright ^cells. Both cell types had low or absent perforin and no granzyme B [8]. In studying the function of immature CD56+ cells, we observed that they had negligible cytotoxicity. Here we describe a novel cell-contact dependent proliferation inhibition of cell lines by cultured CD56+ cells which suggests that immature CD56+ cells may have novel growth regulatory properties.

## Materials and methods

### Antibodies and reagents

Fluoroscein isothiocyanate (FITC)-conjugated anti-CD56, anti-CD16, anti-CD33, anti-CD3, anti-CD2, anti-CD11a, anti-CD94, anti-CD80, anti-CD44, anti-granzyme A, Allophycocyanin (APC)-conjugated anti-CD56, anti-CD11c, anti-CD38, anti-CD69, anti-CD117, (Pe)-conjugated anti-CD117, NKB1 (KIR3DL1), anti-CD3, anti-CD16, anti-CD56, anti-perforin, PerCP-conjugated anti-CD3, anti-CD69, anti-CD8 and matching isotype mouse mAbs were purchased from Becton and Dickinson (S Jose, CA). Pe-conjugated anti-CD34, P58.1 (NK2DL1), P58.2(NK2DL2), NKG2A were purchased from Immunotec (Marseille, France). Magnetic beads-conjugated anti-CD56 and mini Macs magnet were purchased from Miltenyi Biotec (Auburn, CA). (APC)-conjugated anti-CD95 and (Pe)-conjugated anti-CD95L were purchased from Caltag (Burlingame, CA). Hyaluronic acid was purchased from Sigma (S Louis, Mo)

### Cell isolation, activation and expansion

CD34+ cells were positively selected from normal donor G-CSF-mobilized peripheral blood stem cells, (PBSC) counted, and frozen in liquid nitrogen until use. All donors gave written informed consent to donate stem cells in NIH protocols 99-H-0046 and 95-H-0049. Peripheral blood mononuclear cells (PBMC) were separated by Ficoll-hypaque density separation. Cells were cultured in RPMI 1640 supplemented with 10% AB or 10% FCS serum, glutamine (2 mM) gentamicin, hereafter referred to as complete medium. CD34+ cells were cultured in complete medium, in 24 or 12 or 96 U well plates (Costar), for a minimum of 10 to a maximum of 70 days. Cells were stimulated every 5–7 days with SCF (20–50 ng/ml) (Peprotech, Rocky Hill, NJ) with or without IL-2 (200 U/ml) or IL-15 (1–100 ng/ml). To obtain pure NK populations, CD34+ cells stimulated for 15–21 day with IL-2 were stained with a Pe-conjugated anti-CD56 Moab and CD56+ cells were isolated by electronic sorting using an EPICS ALTRA Flow cytometer (Beckman Coulter, Miami, FL). In some experiments, immature CD56+ cells were selected with an anti-CD56 conjugated magnetic bead column (Miltenyi Inc.). Vigorous mechanical pressure eluted the CD56+cells retained in the column. Peripheral blood NK cells were negatively selected by magnetic sorting using a Miltenyi isolation kit. Positively and negatively selected peripheral blood NK cells were further expanded in vitro as follows: 100 μl of 1 × 10^5 ^/ml CD56+ were mixed with 100 μl of 75 Gy irradiated LCL cells in complete medium supplemented with IL-2 (10 U/ml) and 15 % conditioning medium were plated in Costar 96/w round bottom plates. Cells were stimulated every 3 days with CM supplemented with 15% CM for the required time.

### Flow cytometric analysis

In some experiments, cells were stained with Pe-Conjugated anti-CD56 or anti-c-Kit (one color). In other experiments, cells were incubated with FITC-anti-CD56 and Pe-anti-c-Kit (two colors) or a combination of Pe, FITC, PerCP, and APC-conjugated antibodies specific for the desired molecules (four colors). In all cases the cells were stained on ice, for 30 minutes, washed twice, fixed in 1% paraformaldehyde (PFA). For intracellular staining experiments (IC), 1 million cells were first stained with a Pe-conjugated anti-CD56 for 15 minutes at room temperature (RT) in the dark than 2 ml of FACS lysing solution was added to the cell mixture.

After 10 minutes incubation at RT, cells were washed and permeabilized with 0.5 ml FACS perm mix for 10 minutes at RT. Cells were than stained with an anti-Pe-conjugated anti-Perforin Moab and a FITC-conjugated anti-granzyme A moAb or Pe and FITC-conjugated mouse control moAb isotypes for 30 minutes at RT, washed and fixed in 200 ul of 1% PFA. Cells were analyzed by a 4 filters BD FACScan Excalibur. For trans-well experiments, after an initial equilibration (1 hour at 37 C) with complete medium, 200 μl of 5 × 10^4 ^- 1 × 10^5 ^CD34 cell- derived NK cells or IL-2 activated NK cells cultures were incubated in complete medium in the upper trans-well compartment of a 12-transwell plate (Costar, Cambridge, MA) in the presence or absence of 5 × 10^3 ^- 5 × 10^4 ^K562, while 1 ml culture of 5 × 10^3^K562 were cultured in the lower compartment. A 0.4 μm polycarbonate membrane separated upper and lower compartments. After a 2-day incubation, aliquots of 200 μl of K562 cell culture were removed from the lower compartment and labeled with 3H-TdR. Breaking and washing the transmembrane then permitted harvesting the upper compartment. Cells were counted or labeled with 3H-TdR.

### Microcytotoxicity assay

For reverse antibody-directed cell cytotoxicity (ADCC) 6 replicates of 20 μl of effector cells were incubated in a 60-well (40 μl depth) Terasaki plate for 30 m at room temperature in the presence or absence of 5 μl (10μg/ml) 3g8 (anti-CD16). At the same time, FcR+P815 (2 × 10^6^) were incubated in 1 ml of complete medium supplemented with 10 μl of Calcein-AM (Molecular Probes, Junction City, OR) for 30' at 37°C, washed four times and diluted to 1 × 10^5^/ml. After diluting the effector cells, 10 μl of target cells were added, plates were centrifuged and incubated at 37°C for 4 hours. In some experiments, effectors with no mAbs were challenged with K562 cells. A few minutes before scanning the plates using a fluorescent detector 5 μl fluoro-quench was added to each well. The percent of lysis was calculated as follows: 1-(mean test-mean blank)/(mean max-mean blank) ×100.

### Reverse transcriptase polymerase chain reaction

Total RNA was extracted from 1 × 10^6 ^positively selected G-CSF-mobilized peripheral blood CD34+ cells, PBMC, resting or IL-2 activated total peripheral blood CD56+ cells and NK cells using Triazol reagent; complementary DNA (cDNA) first strand was produced using Maloney murine leukemia reverse transcriptase (Roche) with an oligo(dt)^12–18 ^anti-sense primer (Roche). The cDNA of interest was amplified as shown in table I. Amplified fragments were analyzed in 1.5 % agarose gel electrophoresis in the presence of ethidium bromide (Sigma, S Louis, MO).

**Table 1:** List of primers used in this study

	**Sequences**		
		
	Sense	Anti-Sense	Transcript
			
Perforin	cggctcacactcacagg	ctgccgtggatgcctatg	369
Granzyme B	ggggaagctccataaatgtcacct	tacacacaagagggcctccagagt	431
NKp30	cagggcatctcgagtttccgacatggcctggatgctgttg	gatttattggggtcttttgaag	606
NKp44	tacttcaaagtgtggcag	tcacaaagtgtgttcatcatc	751
NKp46	aaaagcaagtgaccatct	aagaacatgcttgttgcagt	337
NKp80	caagatgaagaaagataca	gagaaccatccacccaagt	568
NKG2D	gaaggcttttatccacaa	ttacacagtcctttgcat	761

NKp30 NKG2D sense12, perforin and granzyme B11have been already described

### Proliferation assay

The proliferation of K562 and P815 was measured using the tritiated thymidine incorporation (3H-TdR) assay. The first three U-wells of each horizontal row of 96 well plates were filled with 200 μl of negatively selected NK cells or positively selected peripheral blood CD56+ cells, then 100 μl of cultured cells were serially diluted in the remaining wells previously filled with 100 μl of CM. Later, 1 × 10^4 ^K562 or P815 cells were added to the cell cultures. After 2 days incubation, cells were pulsed with 1 μCi of 3H-TdR per well (Amersham Biosciences, Piscataway, NJ) Eighteen hours later, 3H-TdR was measured using a beta scintillation counter.

## Results

### Kinetics of CD56+ cell generation

**Figure 1:**
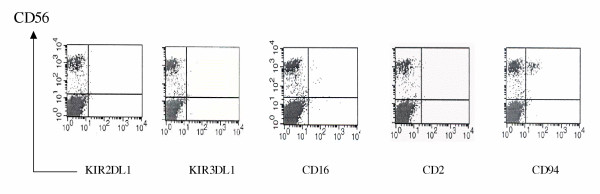
Phenotypic analysis of G-CSF mobilized CD34+ cells cultured in SCF and IL-2 After 3 week stimulation with SCF (100 ng/ml) and IL-2 (200 U/ml), G-CSF mobilized CD34+ cells were stained with for the indicated NK cell surface antigens using specific moAb.

After three weeks stimulation with IL-2 and SCF, G-CSF mobilized peripheral blood CD34+ cells generated CD2-, CD56^high ^(10.6 ± 11.4) (range 3–39 %) and CD56^low ^(3.4 ± 2.0) (range 0.7–5 %) in a sample size of 7 normal donors (REF). Some CD56^high ^cells expressed CD94 and NKG2A but lacked expression of KIR with immunoglobulin like domains KIR2DL2, while CD56^low ^were KIR negative (Figure 1).

### Immature CD56+ cells lack granzyme B and perforin

**Figure 2:**
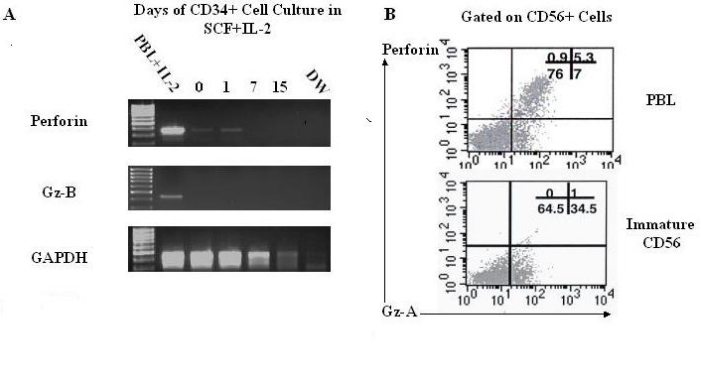
Cytotoxic granule content of immature CD56+ cells: **A**. Absence of perforin and granzyme B in CD34+ cells incubated with IL-2 and SCF for up to 15 days compared with control (representative of three experiments). **B **CD56+ cells from three week cultures showing presence of granzyme A but not perforin compared with control IL-2 stimulated PBL.

We next evaluated the stage of differentiation of molecules associated with cytotoxicity in immature CD56+ cells. Perforin and granzyme B mRNA expression was measured by RT-PCR in CD34+ cells stimulated with IL-2. Perforin was observed on day 0–1 of culture, but disappeared by day 7. Intracellular staining of immature CD56+ cells revealed a granzyme A content comparable to that found in IL-2 activated PBL (Fig. 2). Notably, the reduction of GAPDH band intensity may reflect loss of viability of the cells upon medium term culture.

### Induction of NKp46 in immature CD56+ cells

**Figure 3:**
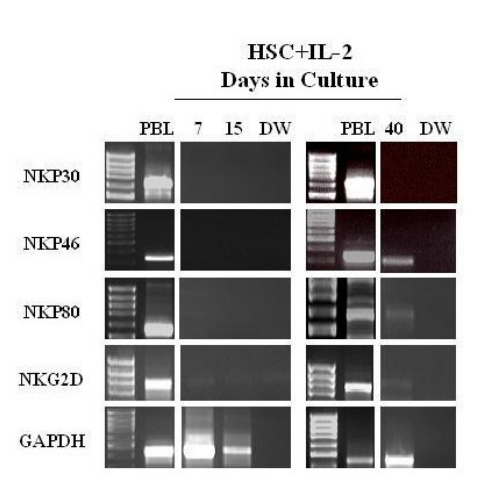
Gene expression of NK activating molecules on CD34-derived CD56+ cells upon stimulation with SCF+IL-2. CD34+ cells were stimulated with SCF and IL-2. At the indicated time, RNA was isolated. NK activating molecules mRNA gene expression was analyzed. By day 15 incubation 39% of CD56+ cells were detected by flow cytometry in the cell culture.

We then sought to determine whether the stimulation of CD34+ cells with IL-2 and SCF induced NK natural cytotoxic receptor (NCR) expression. Within two week of culture, immature CD56+ cells expressed some NK activation molecules including CD44, CD69, and CD38 (data not shown) but did not express NCR genes until at least 6 weeks. At than time CD56+ cells expressed NKp46 but not NKp30. Traces of NKG2D and NKP80 RNA expression were also detected by PCR (Fig. 3).

### Functional assays of CD56+ cells

**Figure 4:**
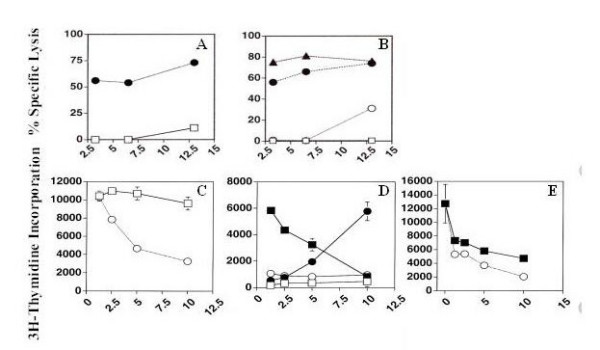
Functional features of immature CD56+ cell upon SCF+IL-2 stimulation. **A**, cytotoxicity of immature CD56+ cells (open squares) compared with IL-2 activated peripheral blood NK cells (closed circles). **B**. Cytotoxicity against P815 cells by immature CD56+ cells incubated with (open circles) or without 3G8 (open squares), compared with peripheral blood NK (closed symbols in the presence (closed triangles) or absence 3G8 (closed circles). This was a representative experiment of series of three. **C **Resting CD34+ cells (open squares) or IL-2 activated peripheral blood NK cells (open circles) were incubated at different ratio with K562. **D**. After 21 days stimulation with IL-2 and SCF, immature CD56+ cells were isolated from whole cells by electronic sorting and incubated in the absence (open squares) or presence (close squares) of K562. IL-2 activated peripheral blood NK cells were cultured in the absence (close circles) or presence (open circles) of K562 (representative of ten experiments). **E**. Inhibition of proliferation of the NK resistant cell line P815 by IL-2 activated peripheral blood NK cells (open circles) or with IL-2 activated peripheral blood NK cells (closed squares). Cells were then labeled with 3H-TdR and radioactivity (CPM) was measured by a β-counter (representative of three experiments).

IL-2 activated immature CD56+ cells showed negligible cytotoxicity against K562 compared with the potent lysis exhibited by peripheral blood NK cells. Similarly in a reverse ADCC assay, immature CD56+ cells coated with an anti-CD16 (3g8) showed only low cytotoxicity against the FcR+ cell line, P815, while control peripheral blood NK cells were strongly cytotoxic. We then investigated the effect of CD56+ cells on proliferation of cell lines. Resting CD34+ cells did not inhibit K562 proliferation, while flow sorted, three week cultured CD56+ cells cultures strongly inhibited K562 cell proliferation in a dose dependent manner, reaching 90% inhibition at an E:T ratio of 10:1. The proliferation inhibition induced by immature CD56+ cells ranged between 29 and 96.5 % (Table 2). Immature CD56+ cells also comparably inhibited the proliferation of the NK resistant cell line P815, indicating that the proliferation inhibition was independent of the resistance of this line to NK-mediated cytotoxicity. Unlike immature CD56+ cells, peripheral blood-derived CD56+ cells proliferated in the presence of IL-2. Nevertheless the proliferation attributable to K562 cells was also abolished when cultured with CD56+ cells (Fig. 4). Trans-well experiments were used to determine whether the inhibition of K562 cell proliferation was mediated by cell-interaction or by soluble factors. In three experiments, the average 3H-TdR incorporation of K562 cells when separated by a membrane from immature CD56+ cells was 5361 ± 1967 versus 5110 ± 1539 cpm for K562 growth in the absence of CD56+ soluble factors. This suggested that proliferation was primarily blocked by cell-cell contact (Table 3). To further examine the mechanism of K562 proliferation inhibition, we cultured K562 with either magnetically sorted immature CD56+ cells in a transwell upper chamber and K562 cells in the lower chamber for two days. Viable K562 cells (> 95% trypan blue negative cells) were recovered from immature CD56+ cells but not from mature CD56+ cultures. After depleting the K562/immature CD56+ cultures of CD56+ cells using antibody-coated magnetic beads, residual K562 cells again proliferated, indicating that in the absence of cytotoxicity, proliferation inhibition was reversible (data not shown).

**Table 2:** CD34-derived CD56+ cells inhibited K562 proliferation

	3H-TdR (CPM)		
		
	ImmatureCD56+ cells + K562	K562 only	% proliferation inhibition
	
1	214	6183.5	96.5
2	267	6183.5	95.7
3	8183	11172	27
4	1805	5283	66
5	1944.5	4720	59
6	743	11232	93.4
7	2708	10323	74
8	355	2524	86
9	138	2524	94.6
10	829	7336	89
Mean ± SD	1718.7 ± 2433.5	6748.1 ± 3255.5	78 ± 22.2

Electronically or magnetically sorted CD34-derived CD56+ cells and incubated 10:1 ratio with K562 and cell proliferation was evaluated after 2-day culture. The difference in the mean CPM incorporation between immature CD56+cells+K562 and K562 alone was highly significant with a P value of 0.0011. The range of CPM incorporation in the presence of immature CD56+ cells and K562 was 138–8103 while in the presence of K562 alone was 2524–11232.

**Table 3:** Transwell Experiments

	3H-TDR (CPM)		
	*Immature CD56	**Mature CD56	***K562
	
Exp 1	6781 ± 51	5941 ± 560	4429 ± 197
Exp 2	3116 ± 65	2534 ± 196	4030 ± 239
Exp 3	6187 ± 512	2932 ± 181	6873 ± 34

Sorted Immature * or mature CD56+ cells** or K562 were incubated 10:1 ratio in the upper chamber at of Transwell plates while K562 were incubated in the lower chamber. After 2 days, lower K562 were collected labeled overnight with 3H-TDR and analyzed in a β-counter 0.4 μm polycarbonate transwell membrane were removed and the upper compartment cells were harvested and evaluated for viability by trypan blue exclusion assay.

## Discussion

Prolonged culture of human CD34+ cells with IL-2 or IL-15, with or without bone marrow stroma cells can generate cytotoxic CD56+ cells [9,5]. These cells are CD94+ and NKG2A+ but it is not clear whether they express other KIRs [9-11]. Here we studied the early stages of NK cell generation from G-CSF mobilized CD34+ cells. After three weeks stimulation with SCF+IL-2, we identified an immature NK population of CD94+ NKG2A+ KIR- CD56+ cells. We previously found that these immature CD56+ populations are heterogeneous. Notably there is a minor subset of CD56^dim ^CD33+ cells that may be precursors to a novel population of CD56+ monocytes [12]. The major CD56+ population with bright CD56+ expression and lymphoid features include a c-Kit+ CD11a- cell and a more mature c-Kit- CD11a+subset. The c-kit+ cell may be the precursor to the c-kit- cell which has lost responsiveness to SCF and has acquired integrins necessary for formation of effector-target conjugates and killing [13]. However, all these CD56+ subsets were immature with respect to functioning cytotoxic apparatus.

Mature cytotoxic NK cells can be generated from CD34+ cells when cultured with an IL-15-producing human spleen fibroblast cell line [14]. However, the generation of fully functional NK cells takes many weeks of cell culture. Using a stroma cell line stimulated with IL-15 Sivori et al did not observe NK mediated cytotoxicity against K562 within the first of month culture. After one month, cells exhibited cytotoxicity but remained KIR negative [15]. Similarly, in our experiments, long term culture induced some mature NK markers – NKp46 and NKp80 gene expression. However within the first month of culture CD34-derived CD56+ cells exerted negligible cytotoxicity. In the absence of any T, B or myeloid cell markers and some NK marker (including NK activation markers CD38, CD44, and CD69), we consider the major population of CD56+ cells to be immature NK cells [16-19]. Their failure to exert cytotoxicity is consistent with perforin and granzyme B knock-out mouse models which lack cell-mediated cytotoxicity, while granzyme A knock-out mice retain cytotoxicity [20-22]. As a consequence, while mature NK cells undergo functional anergy and apoptosis on contact with K562 cells [23,24] mixed cultures of immature CD56+ and K562 maintained cell numbers.

Because immature CD56+ cells produce a variety of cytokines [6], we explored the possibility that they might have functional properties other than cytotoxicity. Remarkably, purified immature CD56+ cells strongly inhibited proliferation of both K562 cells and the NK resistant cell line P815 at low E:T ratios. The absence of detectable perforin excluded the possibility that the effect was due to contamination by a small population of surviving NK cells. Maximum inhibition of proliferation was only seen when effector-target contact occurred. This suggests that immature CD56+ cells, while lacking cytotoxicity, had a novel cell-contact dependent cytostatic effect. Our findings support earlier observations suggesting that bone marrow NK cells regulate hematopoiesis through a non-cytotoxic pathway [25-28]. It is not clear what is the mechanism utilized by immature CD56+ cells for K562 and P815 proliferation inhibition. Cell surface TGF-beta expression on immature CD56+ cells may be responsible for such inhibition since CD34+ cells upon SCF and IL-2 stimulation acquire TGF-beta gene expression (data not shown).

In conclusion, we describe here early stages of NK cell generation from G-CSF mobilized CD34+ cells in the presence of SCF and IL-2. Although immature NK cells lack the cytotoxic apparatus required for classical NK-like cytotoxicity they had the unusual property of inhibiting proliferation of K562 and P815 cell lines. In our future studies we are planning to assess whether the antiproliferative effects of immature CD56+ cells will also involve cells of myeloid lineages and non hematopoietic cell lines. It is possible that these cells normally reside in the bone marrow and have a regulatory effect on hematopoiesis
